# Cross-sectional Average Length of Life Entropy (${\mathcal{H}}_{\text{CAL}}$): International Comparisons and Decompositions

**DOI:** 10.1007/s10680-024-09711-9

**Published:** 2024-07-25

**Authors:** Wen Su, Vladimir Canudas-Romo

**Affiliations:** School of Demography, College of Arts and Social Sciences, Australian National University, Canberra, Australia

**Keywords:** Lifespan inequality, Decomposition methods, Lifespan variation, Life table entropy, CAL

## Abstract

Keyfitz and Leser’s life table entropy was proposed to serve as a relative inequality in mortality measure. Entropy considers the variation around the age at death relative to the length of lifespan in a population, allowing comparisons across time and populations. It is used widely in period and cohort applications. Here, we propose extending this measure and present an index that incorporates the history of survival of all cohorts present at a given time, namely the cross-sectional average length of life entropy, or CAL-entropy (${\mathcal{H}}_{\text{CAL}}$). We decompose cross-population differences of CAL-entropy into the contribution of longevity and lifespan variation, and the change of those differences across time. Our illustrations show that populations are converging regarding lifespan inequality. Lifespan variation holds a noticeable share in the CAL-entropy gap among selected European populations. Longevity held once a pronounced share in CAL-entropy differences and their change, but its influence has receded over the years. The US demonstrates a unique trend where it performs worse across time compared to the selected European populations, and lifespan variation has played a major role in this process. This study signals the importance of lifespan variation in reducing inequality in mortality among developed and longevous populations.

**Supplementary Information:**

The online version contains supplementary material available at 10.1007/s10680-024-09711-9.

## Introduction

In the past, research on mortality focused on studying the average mortality level, often expressed with measures of longevity or length of lifespan (Oeppen & Vaupel, 2002). In recent decades, studies on mortality have extended to include variation around the age at death, or lifespan variation, as a complementary component (Edwards & Tuljapurkar, 2005; Engelman et al., 2010; Gillespie et al., 2014; Shkolnikov et al., 2003; van Raalte & Caswell, 2013; Vaupel & Canudas-Romo, 2003; Wilmoth & Horiuchi, 1999). Life expectancy (${e}_{0}$) as a metric for measuring average lifespan is widely used across multiple disciplines, including political science (Mackenbach, 2013), biology (Colchero et al., 2016), and psychology (Dunkel et al., 2010). Life expectancy is usually synonymous with period life expectancy, which is derived from age-specific mortality rates in a given period (generally a year) and corresponds to a synthetic cohort subject to the mortality experience of that given year (Preston et al., 2001). Similarly, measures of lifespan variation can be calculated based on period life table age-specific functions. For example, e-dagger ($e^{\dag }$) in period life tables refers to the variation in the ages at death of a population at a given time (Edwards & Tuljapurkar, 2005; Gillespie et al., 2014; Shkolnikov et al., 2003; Vaupel & Canudas-Romo, 2003). This addition to mortality research has shed new light on human survival, namely the need to address existent inequality in mortality (or lifespan inequality, hereafter used interchangeably) (van Raalte et al., 2018). Research on longevity measures and lifespan variation, which reflects the uncertainty of death (Vaupel et al., 2011), has been able to provide insights into time dynamics of specific countries/regions, or differences between sexes (Aburto & van Raalte, 2018; Cui et al., 2019). Cohort﻿ life expectancy summarizes the mortality age-profile of a cohort through time to the highest possible age. Its lifespan variation is calculated similarly to period lifespan variation, but using cohort age-specific mortality rates instead.

To assess inequality in mortality, the one frequently applied approach is comparing life expectancy and lifespan variation separately (e.g., van Raalte et al., 2018) or using graphic representations of correlations between longevity and lifespan variation (e.g., Aburto et al., 2020; Permanyer & Scholl, 2019; Vaupel et al., 2011). Through these methods, it is possible to observe that some populations are experiencing reversal and stagnating patterns in lifespan variation (Aburto & van Raalte, 2018; Brønnum-Hansen, 2017; van Raalte et al., 2018). However, the entropy of the survival function which incorporates longevity and lifespan variation brings new possibilities to examine the dynamics of inequality in mortality.

### Entropy

Keyfitz (1977) proposed entropy of the survival function as a measure of the effect on the life expectancy of a proportional change in age-specific mortality, also reflecting the concavity (opposite of rectangularization) of the survival curve in a life table (Ebeling et al., 2018; Keyfitz, 1985; Wilmoth & Horiuchi, 1999). This work was based on Leser (1955) and used by Demetrius (1974, 1978) to describe the variability of the contribution of different age classes to the stationary age distribution and survival process. The Keyfitz–Leser life table entropy, referred to as life table entropy in the rest of the text and denoted by $\mathcal{H}$, is used by demographers to examine the progress of survival improvement in populations (Colchero et al., 2016; Goldman & Lord, 1986; Keyfitz & Golini, 1975; Vaupel, 1986). In recent years, it has also been used to explain how COVID-19’s mortality regime affects period life expectancy and life years lost (Goldstein & Lee, 2020), an original idea that can be traced back to Mitra (1978). Life table entropy entails the property to measure mortality age-distributions, which derives from the similarity to the entropy measure in Shannon’s information theory (Shannon, 1948), assigning values from 0 to 1; 0 signifies that all deaths are concentrated in a single age, and when age-specific death rates are equal across all ages, the life table entropy is 1. However, according to Goldman and Lord (1986), life table entropy could exceed 1 when infant mortality is extremely high, or mortality data is of poor quality. Previous studies have tried to motivate relative indicators of mortality by finding a set of desirable properties that allow for comparisons of lifespan variation across populations with substantially different age-at-death distributions (Bonetti et al., 2024; Carter-Pokras & Baquet, 2002; Shi et al., 2023; Vigezzi et al., 2022). Life table entropy can also be written as lifespan variation ($e^{\dag }$) over life expectancy (${e}_{0}$) (Goldman & Lord, 1986; Hakkert, 1987; Vaupel & Canudas-Romo, 2003), and can be seen as a relative measure of lifespan inequality that can be compared across populations, since the lifespan variation ($e^{\dag }$) in each population is contrasted to its level of life expectancy. This ratio has been explored in mortality modeling to prove the relationship between mortality model parameters for life table entropy and its two components: longevity and lifespan variation (Baudisch & Aburto, 2021; Fernandez & Beltran-Sanchez, 2015; Wrycza, 2014). Furthermore, the change over time in life table entropy can be decomposed to examine the contribution from $e^{\dag }$ and ${e}_{0}$ to its change (Fernandez & Beltran-Sanchez, 2015).

### Cross-Sectional Average Length of Life Entropy

Although period and cohort life expectancy have proven to be valuable in many circumstances, an alternative measure that includes the historical mortality experience and current mortality profile could further strengthen our understanding of inequality in mortality, as it did for longevity (Canudas-Romo & Guillot, 2015; Guillot, 2003). A suggested metric that includes the complete mortality experience in a population was proposed by Brouard (1986; Brouard & Riffe, 2018) with the name “Cohort proportion survival”. Guillot (2003) expanded this idea into the cross-sectional average length of life ($\text{CAL}$). $\text{CAL}$ is a measure that incorporates every life table survival function of the cohorts alive in a given period. Cross-national population differences in CAL can be decomposed by age, cohort, and cause of death (Canudas-Romo & Guillot, 2015; Canudas-Romo et al., 2020; Nepomuceno et al., 2021). $\text{CAL}$ includes survival functions as in period and cohort life expectancy, but incorporates probabilities of surviving for a multitude of cohorts, and therefore serves as a longevity measure incorporating current cohorts’ historical mortality experience (Guillot, 2003). This property gives $\text{CAL}$ an alternative interpretation compared to period or cohort measures, namely, the outlook of a population at a given time. Recently, this comparability was further elaborated by applying e-dagger, an existing measure of the variability of period and cohort life tables to $\text{CAL}$, and deriving the cross-sectional average inequality in lifespan (${\text{CAL}}^{\dag }$) (Nepomuceno et al., 2021). Building on these similarities regarding $\text{CAL}$ and ${e}_{0}$, as well as between ${\text{CAL}}^{\dag }$ and $e^{\dag }$, life table entropy from a $\text{CAL}$ perspective is henceforth studied. The Cross-sectional Average length of Life Entropy (denoted as ${\mathcal{H}}_{\text{CAL}}$, which reads CAL-entropy) is constructed as the ratio of ${\text{CAL}}^{\dag }$ over $\text{CAL}$. CAL-entropy and ${\text{CAL}}^{\dag }$ have complementary mathematical properties, the former as a relative inequality in mortality measure, and the latter as a measure of absolute lifespan variation (see Shi et al., 2023). These further elements of CAL, namely quantifying its relative inequality in mortality, have not yet been explored, and here we present both analytic derivations and empirical applications addressing this. We show that CAL-entropy enables us to compare the lifespan inequality of different populations, and that it serves as a relative lifespan inequality measure.

The study’s main objective is to assess CAL-entropy’s property as a relative inequality in mortality measure, which incorporates the dimensions of longevity and the lifespan variation of all the cohorts present at a given time. CAL-entropy can be compared across populations to find leaders and laggards in lifespan inequality. A decomposition of the comparisons of CAL-entropy is presented, disentangling the contribution of lifespan variation (${\text{CAL}}^{\dag }$) and longevity (*CAL*) to those CAL-entropy gaps. A second decomposition with respect to time helps discern how those components of the CAL-entropy gaps between populations change over time. Together, these decompositions allow us to answer questions concerning diverging trends between populations in terms of lifespan variation, longevity, or both. We illustrate the measure and its decompositions by comparing CAL-entropies between countries and across time.

The rest of the text is structured as follows: The next section describes the data used and the measure proposed, followed by the results which present the illustrations of the comparison of entropy for the period and cohort life tables, and CAL-entropy. Results also include decompositions of differences in cross-population comparisons and their change over time. The last section discusses the implications of the findings and possible future directions.

## Methods and Data

### Data

Data from the Human Mortality Database, HMD (2023) was used to calculate the longevity measures. All populations with historical mortality information long enough to construct the Cross-sectional average length of life and its derived measures (over 30 years, from 1989 to 2018) were included in the study: Denmark, England & Wales, France, Finland, Italy, Netherlands, Norway, Scotland, Sweden, and Switzerland (Iceland was excluded for its small population which created random fluctuations in trends). Life table functions, based on smoothed age-specific death rates from the HMD (Kannisto model at old ages, see HMD protocol), for the selected populations were used to construct the $\text{CAL}$ measures as they show no clear deviations from measures calculated from unsmoothed death rates (sensitivity analysis is shown in the Online Supplement Material, OSM 1). Apart from these selected European populations, we also included data for the United States from HMD combined with mortality estimates from the US Social Security Administration (Bell & Miller, 2005) from 1900 to 1932, which allowed the construction of $CAL$ for 7 years, from 2011 to 2018 (details of the latter data are included in OSM 2).

### Life Table Entropy ($\mathcal{H}$) in $\text{CAL}$ Perspective

The entropy of the life table, denoted ${\mathcal{H}}_{i}\left(t\right)$ with *i* corresponding to period or cohort, has two components: the lifespan variation component, or $e_{0,i}^{\dag } \left( t \right)$; and the longevity component or life expectancy, denoted ${e}_{0, i}\left(t\right)$; as noted by Vaupel and Canudas-Romo (2003):

1 $${\mathcal{H}}_{i} \left( t \right) = \frac{{e_{0,i}^{\dag } \left( t \right)}}{{e_{0,i} \left( t \right)}} = - \frac{{\mathop \smallint \nolimits_{0}^{{\upomega }} \ell_{i} \left( {x,t} \right)ln\left[ {\ell_{i} \left( {x,t} \right)} \right]{\text{d}}x}}{{\mathop \smallint \nolimits_{0}^{{\upomega }} \ell_{i} \left( {x,t} \right)\,{\text{d}}x}},$$

where ${{\ell}}_{i}\left(x,t\right)$ is the life table survival function at age *x* and time *t* for perspective *i* (period or cohort). In the rest of the text, we refer to these as the period ${\mathcal{H}}_{\text{p}}$ and cohort ${\mathcal{H}}_{\text{c}}$ entropy.

Since $\text{CAL}$ shares comparable mathematical structure and properties with period and cohort life expectancies, a similar entropy expression can also be calculated. According to Guillot (2003), the mathematical expression of $\text{CAL}$ is:

2 $$\text{CAL}\left(t\right)={\int }_{0}^{\upomega }{{\ell}}_{\text{c}}\left(x,t-x\right)\hspace{0.17em}dx,$$

where ${{\ell}}_{\text{c}}\left(x,t-x\right)$ is the cohort life table survival function at age *x* for the cohort born at time *t-x*. Building on this equation, Nepomuceno et al. (2021) explored the lifespan variation of CAL, coining it as the cross-sectional average inequality in lifespan (${\text{CAL}}^{\dag }$):

3 $$\begin{array}{*{20}c} {{\text{CAL}}^{\dag } \left( t \right) = - \mathop \int \limits_{0}^{\omega } \ell _{{\text{c}}} \left( {x,t - x} \right)\ln \left[ {\ell _{{\text{c}}} \left( {x,t - x} \right)} \right]\,{\text{d}}x} \\ \end{array}.$$

Based on these two components in the $\text{CAL}$ perspective, we propose to examine the age distribution of mortality of all cohorts present at a given time in a cross-sectional measure, or the CAL-entropy (${\mathcal{H}}_{\text{CAL}}$):

4 $$\begin{array}{*{20}c} {{\mathcal{H}}_{{{\text{CAL}}}} \left( t \right) = \frac{{{\text{CAL}}^{\dag } \left( t \right)}}{{{\text{CAL}}\left( t \right)}} = - \frac{{\mathop \int \nolimits_{0}^{\omega } \ell _{{\text{c}}} \left( {x,t - x} \right)\ln \left[ {\ell _{{\text{c}}} \left( {x,t - x} \right)} \right]\,{\text{d}}x}}{{\mathop \int \nolimits_{0}^{\omega } \ell _{{\text{c}}} \left( {x,t - x} \right)\,{\text{d}}x}}} \\ \end{array}.$$

CAL-entropy has a similar meaning to period or cohort life table entropy: it is maximized with the value of 1, which corresponds to mortality rates equal across all ages and cohorts, and it is minimized with a value of 0, when all mortality is concentrated in one specific age and belonging to one cohort.

**Fig. 1:**
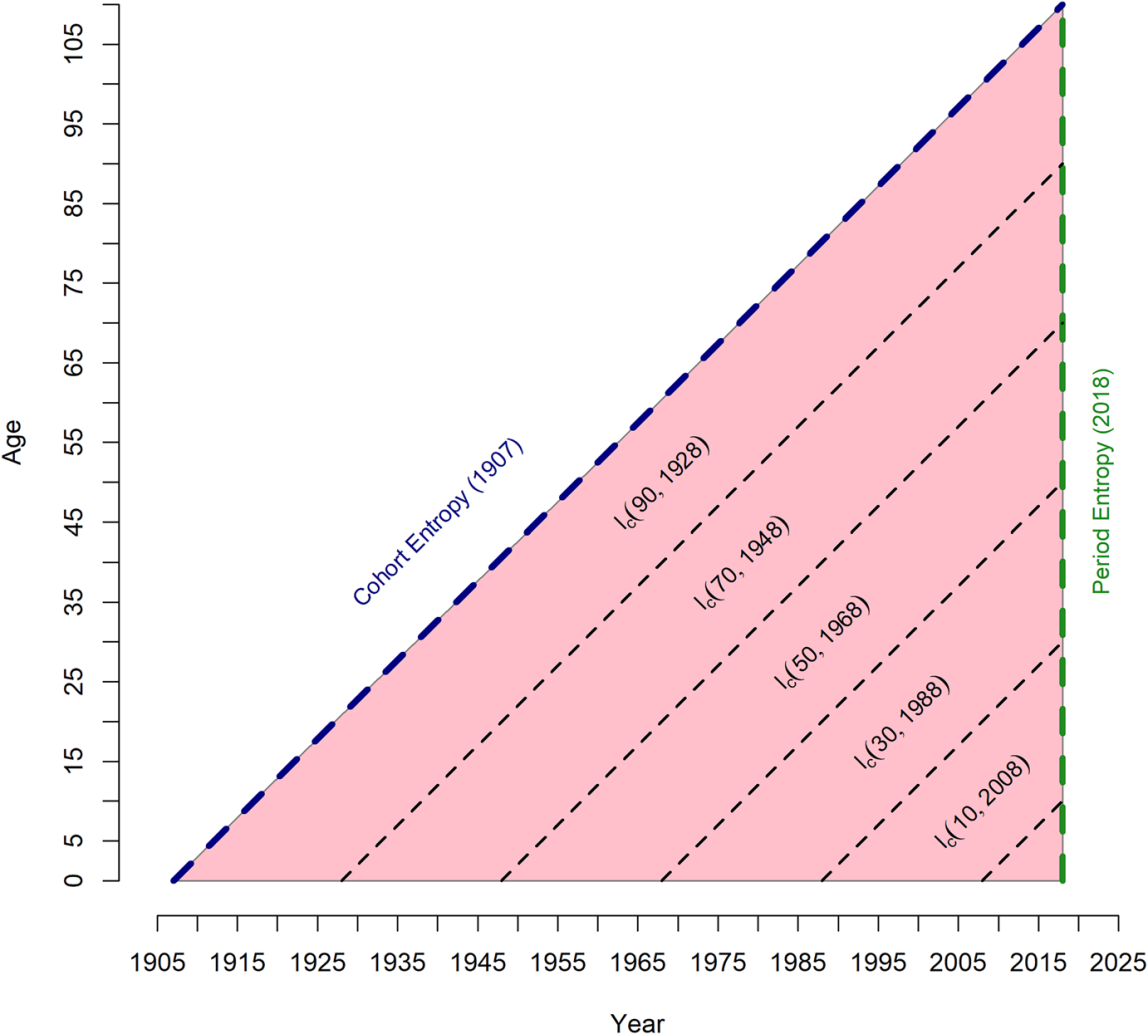
Illustration of the data used by the period, cohort, and CAL-entropy. *Notes* The blue dotted line represents the cohort entropy, and the green dotted line represents the period entropy. The pink triangle and the dark dotted line simultaneously represent the mortality information CAL-entropy comprises

Figure 1 is an illustration of the mortality information CAL-entropy incorporates in comparison with period and cohort entropy. Period entropy measures lifespan inequality from the survival probabilities of a given period (dark dotted green line in 2018), while cohort entropy measures lifespan inequality following the life trajectory of one cohort (dark dotted blue line for the cohort born in 1907 and reaching age 111 in 2018). CAL-entropy, on the other hand, measures lifespan inequality from the survival probabilities of all cohorts present at a given time (2018 in Fig. 1, or all the diagonal lines between the lines of the cohort of 1907 and the period of 2018).

### ${\mathcal{H}}_{\text{CAL}}$ Cross-Population Comparison

An average level of entropy for selected European populations was constructed by taking the arithmetic mean of all their entropy levels, which shows no noticeable difference from an average constructed on age-specific death rates in a sensitivity analysis (see OSM 1). This arithmetic mean is calculated as an unweighted mean, giving the same position to each of the populations included. We will refer to this average level as the selected European populations’ average entropy. The term “selected European populations” encompasses information on the following countries: Denmark, England & Wales, France, Finland, Italy, Netherlands, Norway, Scotland, Sweden, and Switzerland. For each year, this average level of entropy is selected as the reference level and each population-specific entropy is compared to it. The differences generated by this comparison can also be interpreted as the disparities in inequality in mortality with respect to the selected European populations’ average entropy level. There are two reasons for using the selected European average as a benchmark for comparison. Firstly, as a relative measure, comparing individual countries to a general benchmark avoids the complexity of comparing each country in pairs. Secondly, the selected European countries, in general, have a higher life expectancy and lower lifespan inequality compared to the rest of the world, even among developed countries (e.g., the US). Engaging this benchmark offers insights to other countries on reducing lifespan inequality to such a level.

For the detailed decomposition, we assume that ${\mathcal{H}}_{\text{CAL}}$ and all the derived measures are continuous functions of a variable $y$, and then examine the derivatives of ${\mathcal{H}}_{\text{CAL}}$ concerning this variable in order to assess population comparisons. A dot on top of the function corresponds with the derivative of that function with respect to the variable $y$ (Newton’s (1704) dot notation for derivatives). All CAL perspective measures (${\text{CAL}}\left( t \right),{\text{CAL}}^{\dag } \left( t \right)$ and ${\mathcal{H}}_{\text{CAL}}(t)$) are calculated for each of the populations included in our illustration and defined as dependent on variable $y$ as ${\text{CAL}}\left( {t,y} \right),{\text{CAL}}^{\dag } \left( {t,y} \right)$ and ${\mathcal{H}}_{\text{CAL}}(t,y)$, corresponding to population $y$. Building on the work of Fernandez and Beltran-Sanchez (2015), who decomposed the changes in lifetable entropy over time into lifespan variation component and longevity component, we derived CAL-entropy’s decomposition as follows:

5 $$\begin{array}{*{20}c} {{\stackrel{\bullet}{\mathcal{H}}}_{{{\text{CAL}}}} \left( {t,y} \right) = {\mathcal{H}}_{{{\text{CAL}}}} \left( {t,y} \right)\left[ {\frac{{{\text{C}}{\stackrel{\bullet}{\text{A}}}{\text{L}}^{\dag} \left( {t,y} \right)}}{{{\text{CAL}}^{\dag} \left( {t,y} \right)}}} \right] - {\mathcal{H}}_{{{\text{CAL}}}} \left( {t,y} \right)\left[ {\frac{{{\text{C}} {\stackrel{\bullet}{\text{A}}} {\text{L}}\left( {t,y} \right)}}{{{\text{CAL}}\left( {t,y} \right)}}} \right]} \end{array}.$$

The derivative of CAL-entropy with respect to $y$, or cross-population comparison, can be decomposed into: differences in lifespan variation and differences in longevity between the populations, or first and second terms on the right of the Eq. (5), respectively.

For the differences-of-differences decomposition, the mathematical expression including the derivative with respect to time is:

6 $$\begin{aligned} \frac{{\partial \left[ {{\stackrel{\bullet}{\mathcal{H}}}_{{{\text{CAL}}}} \left( {t,y} \right)} \right]}}{{\partial t}} = & \,\frac{{\partial {\mathcal{H}}_{{{\text{CAL}}}} \left( {t,y} \right)}}{{\partial t}}\left[ {\frac{{{\stackrel{\bullet}{\text{CAL}}}^{\dag } \left( {t,y} \right)}}{{{\text{CAL}}^{\dag } \left( {t,y} \right)}} - \frac{{{\stackrel{\bullet}{\text{CAL}}}\left( {t,y} \right)}}{{{\text{CAL}}\left( {t,y} \right)}}} \right] + {\mathcal{H}}_{{{\text{CAL}}}} \left( {t,y} \right)\frac{\partial }{{\partial t}}\left( {\frac{{{\stackrel{\bullet}{\text{CAL}}}^{\dag } \left( {t,y} \right)}}{{{\text{CAL}}^{\dag } \left( {t,y} \right)}}} \right)~{~} \\ & - {\mathcal{H}}_{{{\text{CAL}}}} \left( {t,y} \right)\frac{\partial }{{\partial t}}\left( {\frac{{{\stackrel{\bullet}{\text{CAL}}}\left( {t,y} \right)}}{{{\text{CAL}}\left( {t,y} \right)}}} \right) \\ \end{aligned} $$

The right side of (6) has three terms for the changes over time in the CAL-entropy gap between each population and the selected EU populations’ average level. The first term captures the contribution of changes in the average entropy across time, referred to as benchmark average entropy. The second and third terms capture the contribution of lifespan variation and longevity. The decomposition of differences in CAL-entropy into the longevity and lifespan variation component is also reminiscent of the variance decomposition used in lifespan inequality research (Caswell, 2023; Edwards, 2011; Permanyer & Scholl, 2019; Smits & Monden, 2009; van Raalte et al., 2012). The variance decomposition separates the variance into additive between-group and within-group components, which can be related respectively to the longevity and lifespan variation differences in our decomposition. However, we prefer the use of longevity and lifespan variation of our decomposition, as they both have intuitive demographic interpretations.

Intuitively, the decomposition method tracks differences in CAL-entropy across populations and separates them into: the effect of gaps in mean lifespan (longevity contribution); and gaps in the variation of age at death (lifespan variation contribution). For example, two populations could have the same CAL-entropy of 0.2, however, one could come from having a low mean longevity of 50 $\left( {\frac{{{\text{CAL}}^{\dag } \left( t \right)}}{{{\text{CAL}}\left( t \right)}} = \frac{10}{{50}}} \right)$, while the other could come from having a substantial lifespan variation of 15 $\left( {\frac{{{\text{CAL}}^{\dag } \left( t \right)}}{{{\text{CAL}}\left( t \right)}} = \frac{15}{{75}}} \right)$. The decomposition of CAL-entropy would disentangle whether the differences between those populations are due to differences in longevity or lifespan variation. In the difference-of-differences decomposition in Eq. (6), the population differences are studied over time and further disentangled into changes in: the mean lifespan differences (longevity component); the variation of age at death (lifespan variation component); and the changes in the benchmark average entropy level, in our case the selected European average. Coming back to the example of the two populations, the narrowing or widening of the differences between the two populations’ CAL-entropy levels could be entirely due to one of the three components mentioned above, or a combination of them. Our decomposition seeks to explore this dynamic.

In our illustrations, comparisons between period and cohort lifetable entropy, and CAL-entropy perspective are shown. We calculated the slopes of the best fitting lines for different entropy perspectives, and the mean intrinsic rate of change across time periods (analogous to the instantaneous growth rate calculations in Preston et al. (2001, p. 9), to compare the speed of change for specific components and populations’ time trends. The share of a specific component’s contribution is assessed as the proportion of that specific component over the total absolute contribution by all components involved. We report the shares in rolling means with respect to total changes to avoid outliers from specific years. All results presented were calculated in R (R Core Team, 2023).

The R code used in the analysis can be accessed at: https://osf.io/mg5p4/?view_only=1c2dc8f0ae6b4f13b9636f2290549cb4

The Shiny interactive application allows the reader to look at results in greater detail for all selected populations and for both females and males, available at: https://demo221.shinyapps.io/Shinyapp-CALentropy/

## Result

### Comparison Between ${\mathcal{H}}_{\mathbf{C}\mathbf{A}\mathbf{L}}$, ${\mathcal{H}}_{\mathbf{p}}$, and ${\mathcal{H}}_{\mathbf{c}}$

**Fig. 2:**
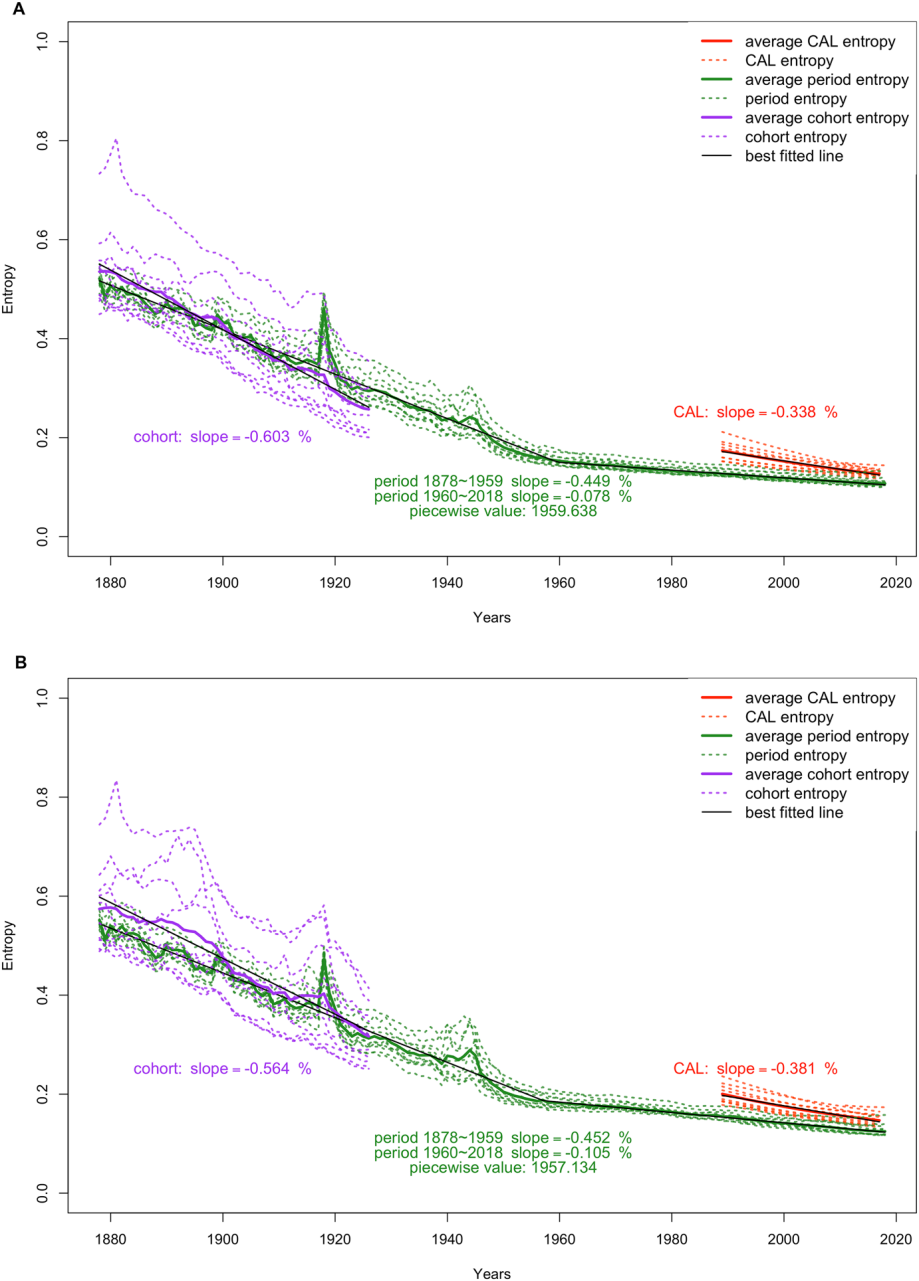
Time trends in the period, cohort, and CAL-entropy for females (2A) and males (2B) in selected populations, 1878–2018. *Notes* Red, green, and purple stand for CAL-entropy, period entropy, and cohort entropy, respectively. Period entropy is fitted with piecewise regression. The piecewise point for period entropy is captioned under its slopes. *Source*: Author’s calculation based on HMD (2023), and for the USA also Ben and Miller (2005)

Figure 2 shows the time trends of the three measures for female and male selected populations from 1878 to 2018: the period and cohort entropy (${\mathcal{H}}_{\text{p}}$ and ${\mathcal{H}}_{\text{c}}$), and the CAL-entropy (${\mathcal{H}}_{\text{CAL}}$). Regression lines are fitted to show the pace of improvements across time (${R}^{2}$=0.99 for all lines). For period entropy, the two big spikes correspond to the 1918 Spanish flu and the years 1940–1945 for World War II. Apart from that, the overall trend for period entropy trajectories was declining, indicating mortality distribution for each year had become further concentrated in fewer ages through time. The paces of improvement in period entropy, or decreases in entropy, for females and males are similar (around 0.45% decline per year) before their respective turning points (females in the year 1959 and males in 1957), changing afterward to a slower pace of improvement for males (decreasing 0.10% per year) and females (decreasing 0.08% per year). Cohort entropy improves at a faster pace than period entropy for both females and males, although females catch up with period levels much faster than males. The pace of improvement for cohort entropy is 0.60% per year for females and 0.56% for males. This phenomenon between cohort entropy and period entropy is due to cohort entropy corresponding to the year of birth, while cohorts benefit from improvements in mortality during their lifetime, and therefore the trajectory of ${\mathcal{H}}_{\text{c}}$ moves at a faster pace. CAL-entropy, compared to the other two measures, incorporates the mortality experience of every cohort present at one point in time and it is smoother than the other two. Since the ${\mathcal{H}}_{\text{CAL}}$ trajectories between females and males do not differ greatly, only cross-population comparisons and decompositions for females are included in the remaining result section, and for males the results can be found in the OSM 3 and the Shiny app.

### Cross-Population Comparison

**Fig. 3:**
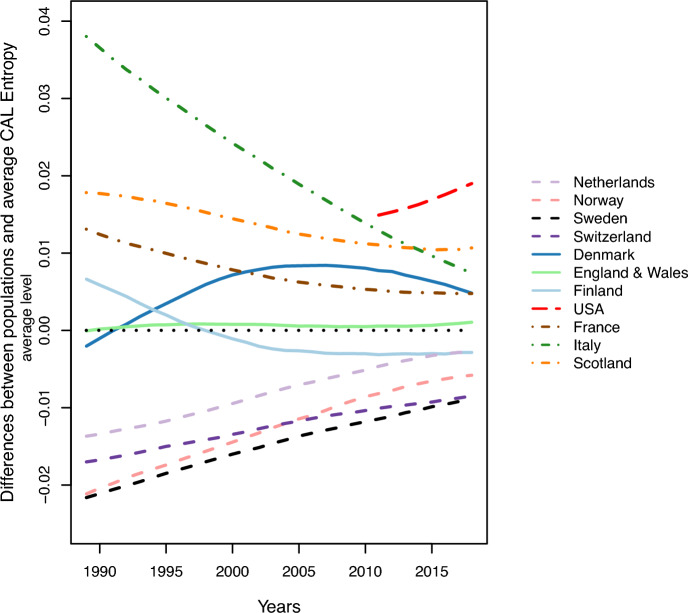
Time trends in differences between population and selected European populations’ average CAL-entropy, female, from 1989 to 2018. *Notes* The horizontal dotted dark line at zero represents the selected European populations’ average entropy level. The solid, dashed and dot-dash lines represent the population that crosses the average entropy, and with more and less heterogeneous mortality distribution than the average entropy level, respectively. *Source*: Author’s calculation based on HMD data (2023) and Bell and Miller (2005)

Figure 3 shows the time trends of absolute differences between each female population’s ${\mathcal{H}}_{\text{CAL}}$ from the selected European average. Over time all populations’ trajectories converged to the average as observed in the narrowing gaps between lines across time, except for the US. However, in recent years, the trend for Scotland, France, Finland, and Switzerland is leveling off with respect to the average level. The trend shows the absolute differences in CAL-entropy reduction (the standard deviation among all the absolute differences in 1989 declines from 0.019 to 0.007 in 2018). Among selected European populations, the Netherlands, Norway, Sweden, and Switzerland started from a more advantageous position compared to the selected European average CAL-entropy and converged to the average level, although at different intensities from country to country; Denmark, England & Wales, and Finland experienced a crossover to the other side of the average level; for the former two populations the heterogeneity of mortality distribution became relatively higher; and for Finland, the cross-over from the average signifies a more compact mortality distribution. ${\mathcal{H}}_{\text{CAL}}$ in France, Italy, and Scotland narrowed its gap with respect to the average level.

Because these trends have similarities among different populations, we will group them by their trajectories. These trends can be grouped into four categories of populations: low inequality (Netherlands, Norway, Sweden, and Switzerland); crossover (Denmark, England & Wales, Finland); improving (France, Italy, and Scotland); and widening inequality population (the United States). The latter three group labels are based on their trajectories. The populations maintaining an advantage with respect to the selected European average, i.e., having lower inequality than average during the entire study period, are labeled “low inequality” countries. We compare the US CAL-entropy level from 2011 to 2018 to the selected European average level. The dashed red line in the upper right corner shows that the US failed to keep up with the selected European populations’ rates of improvement in reducing heterogeneity in mortality across ages. The US is categorized as a widening inequality population (also see e.g., Rogers et al., 2020; Sasson, 2016; Wrigley-Field, 2020).

### Decomposition of CAL-Entropy

**Fig. 4:**
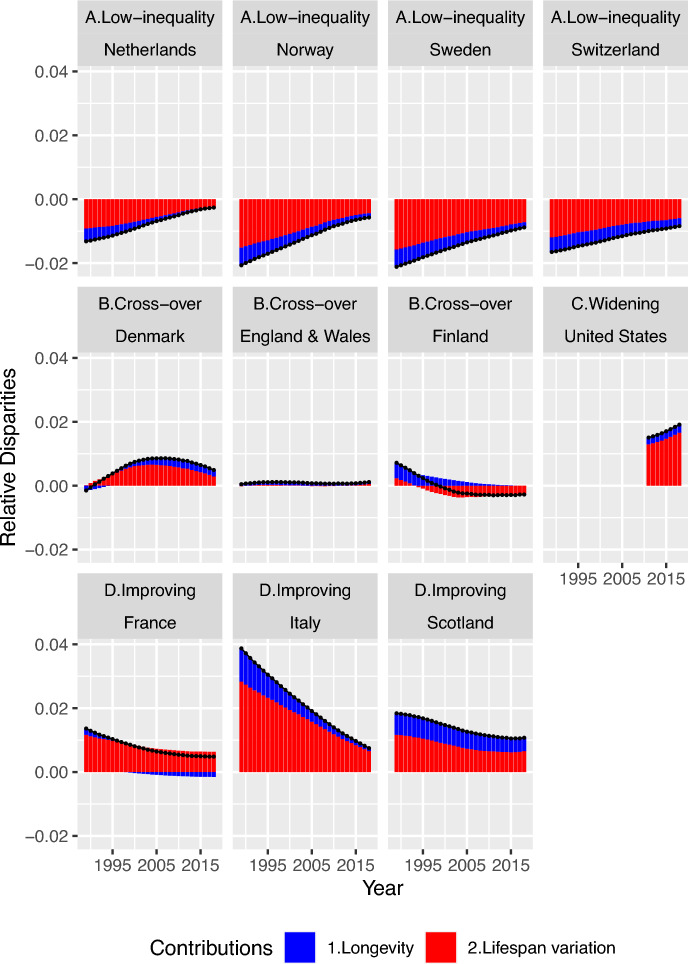
Decomposition of the female CAL-entropy gap between the selected European populations’ average and specific populations into longevity and lifespan variation, 1989–2018. *Notes* red and blue stand for contributions from changes in lifespan variation and longevity, respectively. *Source*: Author’s calculation based on HMD (2023) and Bell and Miller (2005)

**Fig. 5:**
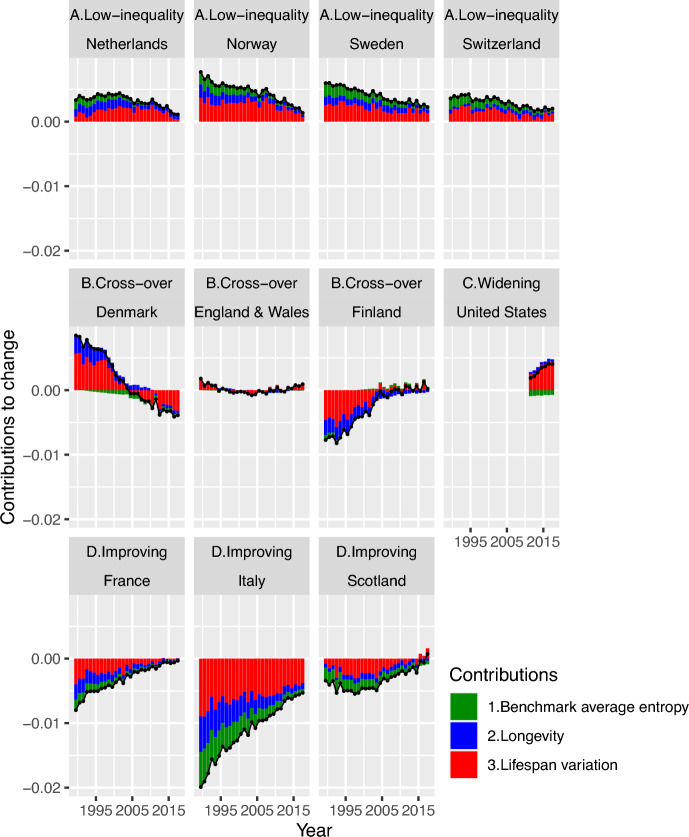
Decomposition of the time changes in female CAL-entropy gap between the selected European populations’ average and specific population across time into average entropy improvements, longevity, and lifespan variation, 1990–2018. *Notes* Green, red, and blue stand for contributions from changes in average entropy level, and from lifespan variation and longevity, respectively. Contributions are multiplied by 100. *Source*: Author’s calculation based on HMD (2023) and Bell and Miller (2005)

Figures 4 and 5 are closely related showing population-specific results, with one describing differences and one describing changes in that difference. Our results are presented by individual populations for the two decompositions.

Figure 4 depicts the contribution of lifespan variation and longevity to differences in entropy between each population and the selected European average (as in Eq. 5). The line and dot represent the total contribution, red bars stand for the contribution from lifespan variation, and blue bars stand for the contribution from longevity. If the bars or lines and dots are below zero, this means longevity and lifespan variation contribute to the reduction of the entropy gap between the individual population and the selected European average. Elements above zero correspond to the exacerbation of the entropy disparity between the individual population and the selected European average. Meanwhile, in order to explore the underlying mechanism and time dynamics that drive the change in CAL-entropy gap, Fig. 5 shows the decomposition of changes over time in CAL-entropy gap between the selected European average and each population (as depicted in Eq. 6 for differences-of-differences). In Fig. 5, bars represent the changes in the corresponding adjacent bars in Fig. 4. For example, the first bar in Fig. 5 for Denmark represents the changes between the year 1989 and 1990; we denote this bar as the year 1990 (or the change from 1989 to 1990 observed in 1990). The bars below zero on the vertical axis represent the contributions that help close the gap toward the selected European average level across time, while the bars above zero signify contributions to an increase in entropy disparities across time, with respect to the selected European average level.

For the low inequality populations (Netherlands, Norway, Sweden, and Switzerland), the trajectory and shares of contributions from both lifespan variation and longevity components to the differences from the selected European average are similar. They are all reducing their differences from an advantaged position in CAL-entropy gap between each population and the selected European average. However, they approach this selected European average level (zero on the y-axis) at a different pace from country to country. For Switzerland, the contribution from longevity is declining much more slowly than for the other low-inequality populations (declining with − 0.02 per year compared to − 0.04 per year for Norway and Sweden), while the Netherlands has a much faster decline in contribution from lifespan variation (− 0.11 per year). On the other hand, with regard to the changes across time, the low-inequality populations have a unifying pattern: the share of longevity component and benchmark average entropy component kept decreasing throughout the years. At the same time, lifespan variation components contributed a steady yet increasing share to total changes in cross-population differences throughout 1990–2016 (increasing from 28 to 51% in total changes for the Netherlands and showing similar trends for the other three populations).

For the CAL-entropy gaps among the Cross-over populations (Denmark, England & Wales, Finland), Denmark had lower CAL-entropy than the selected European average level at first, but then it moved from below- to above-average values, and in doing so reached a more heterogeneous mortality age distribution. Throughout this process, which spans from 1989 to 2003, a large proportion of the positive contribution comes from lifespan variation, while longevity opposes this trend in the initial years. During this time, the Danish longevity component maintains a share of around 36% in total changes alongside lifespan variation, which reduces from 64 to 29% in the process of crossing over and then shifting away from the average. However, around the year 2004, the process slowed down, and changes in benchmark average entropy and longevity dominated the turning point (57% of the total change). After that, Denmark started approaching the average level again; along this process, lifespan variation regained its dominant position over the other two components (with around 80% of the total change). This signifies that Denmark’s efforts to reduce the level of lifespan variation have made an impact. England & Wales does not have a pronounced gap with the average level, therefore its contribution is less apparent. For Finland, the story is the same as Denmark, with longevity and lifespan variation contributing to improvements in closing the entropy disparities. Differences in the longevity component contributed to a large part of Finland’s disparities from the selected European average in CAL-entropy gap before 1998. After that year, the changing contribution of lifespan variation became the driver of the lower CAL-entropy compared to the selected European average. When looking at the changes across time, after 2002, the level of contributions to time changes remained at a steady level until recently, leaving virtually no pronounced contribution from any component. This means Finland has maintained a constant improvement in inequality in mortality, keeping a similar pace of improvement with the average level but at a position of lower CAL-entropy compared to the selected European average (see Fig. 5).

The US is expanding its CAL-entropy disparities compared to the selected European populations’ average level, and a large portion of this increasing relative disparity is the contribution from lifespan variation. The US shows a consistent upward trend of total contributions to change in CAL-entropy, with the lifespan variation component holding the major role (67–77% of total contributions) in the widening gap between the country and the selected EU average.

As for improving populations (France, Italy, and Scotland), France’s entropy disparities were driven by contributions from lifespan variation, which is noticeably higher compared to the selected European average, while its contribution from longevity has partially offset those effects by having higher longevity levels than the selected European average since 1999. Lifespan variation and longevity have grown to a relatively stable share across time for Scotland (changes from 36 to 40% of the total contribution for longevity, and around 63–59% for lifespan variation). For Italy, as its CAL-entropy levels are fast approaching the average level, the share of longevity is growing smaller and smaller (changes from 25 to 12% of total contributions in differences from the selected European average). For changes across time, the Finnish pattern of constant improvements holds for the improving populations of France and Scotland, with the difference leveling off at a certain level with respect to the selected European average. Italy’s contribution from lifespan variation has been steady, with the contribution from the longevity and benchmark average entropy shrinking over time (from 55% of the total changes to 29%).

Overall, contributions from lifespan variation are still a major part of the driving force in entropy differences and time changes in entropy gaps for most low-mortality populations. Longevity components and benchmark average entropy, represented by the change in average level, decrease across the years with little sign of resurgence.

## Discussion

In this study, cross-population comparisons using CAL-entropy were assessed. This enabled us to understand how two important components, lifespan variation and longevity, determined the CAL-entropy gap among populations. The $\text{CAL}$ perspective differs from the traditional period or cohort perspective in the interpretation and mortality information utilized (Guillot, 2003; Nepomuceno et al., 2021). Compared to traditional life table entropy (Keyfitz, 1977; Leser, 1955), CAL-entropy is more robust and less sensitive to period shocks and offers a complementary perspective that measures the lifespan inequality of a population. For example, COVID-19 has greatly affected some of the studied populations, particularly the USA (Aburto et al., 2021; Schöley, et al., 2022). Entropy in the period perspective will be greatly affected by this excess mortality during the pandemic. On the contrary, CAL-entropy is likely to show small changes from the results presented here, given that it includes all of the mortality experiences of all cohorts present at a given time. This robustness of the results highlights the relevance of the CAL perspective to study inequalities in age at death. CAL-entropy cannot replace the timely role of the period entropy measure, or the uniqueness of the cohort entropy for births followed over their entire lifespan. However, by incorporating all the cohorts present at a given time in its calculation, CAL-entropy makes a realistic estimation of how lifespan inequality unfolds in the entire population, as opposed to one time or one cohort. This alternative perspective can reveal persistent and emerging trends regarding populations as a whole.

For selected European populations, a converging time trend toward the average level among the populations selected was observed on both an absolute and a relative scale. This could indicate that the disparities in developed countries are narrowing with progress in mortality improvement at younger ages, which translates into a more concentrated mortality distribution around the modal age at death (Canudas-Romo, 2010; Horiuchi et al., 2013; Ouellette & Bourbeau, 2011; Vaupel et al., 2021) This observation coincides with a number of previous studies on the upward trend of equality in mortality through human history (Colchero et al., 2016; Fernandez & Beltran-Sanchez, 2015; Nagnur, 1986; Nemeth, 2017; Nusselder & Mackenbach, 1996). This phenomenon may be attributed to the fact that all the selected European populations and the United States went through epidemiological transitions around the same time and shared similar economic advancements (Marinho et al., 2013; Omran, 2005). However, it also means that the group of countries denoted as low-inequality populations are not keeping their initial advantage with respect to the average level across time. For most low-inequality populations, this trend is a combination of smaller longevity improvements compared to the selected European average, and the fact that the average longevity is catching up at a faster pace. CAL-entropy differences in France and Scotland from the selected European average have reached a plateau in recent years despite making improvements in longevity. In other words, the longevity improvements in France and Scotland did not entirely offset the disparities concerning the lifespan variation component with respect to the selected European average. Specifically, the relatively faster increase in longevity was accompanied by a stalling or slower pace of lifespan variation decrease for France and Scotland (Seaman et al., 2019), and therefore created a stable or even widening disparity compared to the selected European average level.

As our CAL-entropy decomposition results have shown, longevity differences among populations are shrinking or becoming stable over time. Longevity has become a less pronounced driving force in CAL-entropy differences among all populations presented, even though in some populations it had a major contribution in the past. The trend could suggest that these populations in low-mortality settings are reaching a similar pace of improvement in longevity (Avendano & Kawachi, 2014; Leon, 2011). On the other hand, the heterogeneous pace of change in lifespan variation across countries dominates the differences in CAL-entropy (Caswell, 2023; Hiam et al., 2021; Timonin et al., 2016). For almost all populations presented in this paper, the entropy gap between specific populations and the selected European average has a pronounced lifespan variation component compared to the longevity component. Lifespan variation differences are taking over the differences in CAL-entropy among populations, except for Scotland, where those shares are halved with longevity (see Fig. 4). Our second decomposition analyzes, details the time changes in CAL-entropy gap between the selected European average level and all the studied populations. Again, the lifespan variation component here leads among the three components. This dominance of the lifespan variation can be explained by looking at the age- and cohort-decomposition of CAL-entropy as done for ${\text{CAL}}^{\dag }$ earlier (Nepomuceno et al., 2021). In the OSM Sect. 5, we have included age- and cohort-decomposition for Italy to show this. The dominance of lifespan variation across populations is partially due to the diverging trend in mortality at younger ages, and older cohorts across populations, to which lifespan variation is sensitive (Aburto et al., 2020; van Raalte & Caswell, 2013; Zhang & Vaupel, 2009). The dominant contributions to the lifespan variation, at younger ages and older cohorts, are likely the accumulation of inequalities in mortality across cohorts that the period measure fails to capture.

Similar to the conclusion made in other studies, reducing differences in lifespan variation, especially premature deaths across populations, is the new challenge to narrow the gap in populations’ health among high-income countries (Aburto & van Raalte, 2018; Permanyer & Scholl, 2019; van Raalte et al., 2018; Vaupel, 2010). This increasing share of lifespan variation could also stem from subpopulations with mortality experiences from different education levels, socioeconomic status or different ethnicities (Brønnum-Hansen, 2017; Sasson, 2016; Sasson & Hayward, 2019; van Raalte et al., 2013; van Raalte et al., 2018). As such, more focus should be put on monitoring heterogeneity reductions within and between population groups (Permanyer & Scholl, 2019; van Raalte et al., 2018; and Vaupel & Yashin, 1985).

This result is moreover related to the dynamics of mortality compression and shifting (Basellini et al., 2019; Bergeron-Boucher et al., 2015). The diminishing share of longevity changes that drive the CAL-entropy gaps among populations is also reflected in the similar pace of the shifting mortality schedule across populations. At the same time, mortality compression (which was present in the past increases in life expectancy, but less so now), together with the shifting mortality process, both affect lifespan variation (Appendix of Bergeron-Boucher et al., 2015).

Our findings show that the US was falling behind with a widening disparity compared to the selected European average. Lifespan variations contribute greatly to the composition of CAL-entropy disparities and serve as the driving force in changes of disparities. The gap in entropy and the contribution from lifespan variation coincides with a large amount of research conducted on the topic. Literature has shown clear differences in age-at-death distribution for the US compared to other selected European countries (Ho & Preston, 2010; Ho & Hendi, 2018; Preston & Vierboom, 2021). This may be due to the excess external causes of death and the racial disparities of age-specific mortality in America, which further adds to the increasing component of lifespan variation (Aburto et al., 2019, 2022; Avendano & Kawachi, 2014; Rogers et al., 2020; Sasson, 2016; Wrigley-Field, 2020).

We have shown that CAL-entropy is a robust, and complementary measure quantifying the inequality in mortality. Decomposing the time changes of differences of CAL-entropy for different populations and the driving forces to the change in those differences, proved that lifespan variation is the vital factor in CAL-entropy time dynamics. The sensitivity of CAL-entropy to a specific cohort and age is yet unclear. Only Nepomuceno et al. (2021) explored this in ${\text{CAL}}^{\dag }$ by separating death distributions in a CAL perspective and CAL at each age. However, this remains to be further investigated in CAL-entropy**.** When studying CAL, one crucial shortcoming is its demand for extensive mortality data. For developed and developing countries with limited mortality data, the truncated cross-sectional average length of life, or TCAL, could become convenient, as it can be calculated even for populations without complete mortality history for every cohort. With cross-population comparison, if all populations have the same cut-off year, then the estimation could still reveal pivotal information about inequality in mortality (Canudas-Romo & Guillot, 2015). Life table entropy is a summary measure that combines effects from all ages and causes of death. Further emphasis can be put on the contribution from premature ages and old ages, or the age-composition of the CAL-entropy differences. Cause deleted life table entropy could also be explored in-depth in the future (Keyfitz, 1977; Vaupel & Canudas-Romo, 2003). Our results are presented separately by sex. Although the general trend observed in females is largely similar to males, females for all populations selected consistently exhibit smaller values compared to males. Regarding our decomposition results, most male populations have similar patterns of contributions as their female counterpart from longevity and lifespan variation to the CAL-entropy gap and its changes over time. Nevertheless, certain populations, such as Finland and France, display distinct dynamics of these components between females and males. Further research that specifically explores sex or gender differences in CAL entropy, or examines the contributions to changes in CAL entropy, while considering both sexes and various gender as subpopulations, would significantly expand the applicability of our methodology in mortality research.

## Conclusion

CAL-entropy incorporates information on the historical mortality experience of populations. This attribute provides an alternative and fruitful insight into the mortality experience of a population and the cohorts it includes. It can also be compared to the traditional period or cohort life table entropy. Furthermore, the cross-population decomposition of CAL-entropy prove useful to assess populations’ differences and time dynamics between longevity and lifespan variation, two vital components of inequality in mortality.

## Supplementary Information

Below is the link to the electronic supplementary material.

Supplementary file1 (PDF 1206 KB)

## Data Availability

The R code used in the analysis can be accessed at: https://osf.io/mg5p4/?view_only=1c2dc8f0ae6b4f13b9636f2290549cb4. The publicaly available Human Mortality Database data can be accessed at: www.mortality.org.
