# Correction: Cross-sectional Average Length of Life Entropy $({\mathcal{H}}_{{{\text{CAL}}}} )$: International Comparisons and Decompositions

**DOI:** 10.1007/s10680-024-09715-5

**Published:** 2024-08-12

**Authors:** Wen Su, Vladimir Canudas-Romo

**Affiliations:** School of Demography, College of Arts and Social Sciences, Australian National University, Canberra, Australia

**Correction to: European Journal of Population (2024) 40:25** 10.1007/s10680-024-09711-9

In this article, published online 7 June 2024, the label in Figure 1 read ‘Chohort Entropy (1907)’, but should have read ‘Cohort Entropy (1907)’.

The original article has been corrected.

