# Supplementary material for: Cross-sectional Average Length of Life Entropy (ℋCAL): International Comparisons and Decompositions
Source: Eur J Popul. 2024 Jul 25;40(1):25. doi: 10.1007/s10680-024-09711-9 (PMC11282051; doi:10.1007/s10680-024-09711-9)
Supplement: Supplementary file 1 — Supplementary file1 (PDF 1206 KB) [file 10680_2024_9711_MOESM1_ESM.pdf]

# Online Supplementary Materials

**OSM 1 – Calculation of the Decompositions** ........ 2

**OSM 2 – Data availability and Estimation of US mortality data before 1933** ........ 4

**OSM 3 – Figures for Male Part** ........ 6

**OSM 4 - $CAL$ Entropy and its sensitivity** ........ 7

**OSM 5 – Age- and cohort-decomposition** ........ 8

**OSM 6 – Life table entropy surface** ........ 9

**Reference:** ........ 11

## OSM 1 – Calculation of the Decompositions

When calculating $CAL$, we use the method:

$$CAL(t) = \int_0^{\omega} \ell_c(x, t-x)dx, \qquad \text{(A1)}$$

with discrete approximation detailed in Guillot (2003), and $CAL^{\dagger}$ is based on Nepomuceno et al. (2021):

$$CAL^{\dagger}(t) = -\int_0^{\omega} \ell_c(x, t-x) \ln[\ell_c(x, t-x)]\, dx, \qquad \text{(A2)}$$

where $\ell_x^c(t-x)$ is the cohort survival function calculated as:

$$\ell_x^c(t-x) = e^{-\int_0^x \mu(a, t-x+a)da}. \qquad \text{(A3)}$$

Sensitivity analysis was carried out using unsmoothed and smoothed age-specific mortality rate from HMD, and the difference generated in $CAL^{\dagger}$ was minor (around -0.0001 to 0.00001 for Sweden in years 1989 - 2017).

Based on Vaupel and Canudas-Romo (2003), we assume the rate of change is constant, therefore the discrete form of the derivative regarding $y$ will be written as follows, taking the example of Sweden and the Western European population average level (referred to below as average):

$$\acute{v}(t, \xi) \approx \frac{ln\left[\frac{v(t,SWEDEN)}{v(t,AVERAGE)}\right]}{h}. \qquad \text{(A4)}$$

The Western European population averages, constructed with the average mortality rate of the population selected and mean probability of survival at each age of the populations selected, show no significant difference in the sensitivity analysis (ranging approximately from 0.001 to 0.0004).

When we are calculating derivative with respect to time using equations 5 and 6, the equations are as follows, according to Vaupel and Canudas-Romo (2003), as well as Preston et al. (Preston et al., 2001):

$$v(t + h/2, y) \approx v(t, y)e^{(h/2)\frac{\ln\left[\frac{v(t+h,y)}{v(t,y)}\right]}{h}}, \qquad \text{(A5)}$$

and

$$\frac{\partial[v(t+h/2,y)]}{\partial t} \approx \frac{\ln\left[\frac{v(t+h,y)}{v(t,y)}\right]}{h} v(t + h/2, y), \qquad \text{(A6)}$$

with $y$ representing change across countries, and $t + h/2$ referring to the mid-point between time $t$ and $t + h$.

We only use these two equations below, which assume linear changes, when $\frac{v(t+h,y)}{v(t,y)} < 0$:

$$v(t + h/2, y) \approx \frac{v(t+h,y)+v(t,y)}{2}, \qquad \text{(A7)}$$

$$\frac{\partial[v(t+h/2,y)]}{\partial t} \approx \frac{v(t+h,y)-v(t,y)}{h}. \qquad \text{(A8)}$$

Among all the estimations of components for males and females from selected populations, approximately 19% (17 out of 88) used the latter method.

**OSM 2 – Data availability and Estimation of US mortality data before 1933**

| Population in HMD | Available Years |
|---|---|
| Denmark | 1835-2020 |
| Finland | 1878-2020 |
| France | 1816-2018 |
| Italy | 1872-2018 |
| Netherlands | 1850-2019 |
| Norway | 1846-2020 |
| Sweden | 1751-2020 |
| Switzeraland | 1876-2020 |
| England & Wales | 1841-2018 |
| Scotland | 1855-2018 |
| US | 1933-2019 |

The HMD database only has US mortality data from 1933-2018. In order to complete the US series from 1900 to 1932 between ages 0-33, to construct a truncated cohort life table for our calculation of $CAL$, mortality data from the Social Security Administration, SSA (Bell & Miller, 2005) was used.

Data on mortality from SSA was available for single ages for the periods and cohorts of 1900, 1910, 1920, and 1930, and for infant mortality, all separated by sex. The single age probabilities of dying were the same for period and cohorts; for example, the probability for the cohort of 1908 at age 1, denoted ${}_1q_1^c(1908)$, is equal to the probability for the period of 1909 at age 1, denoted ${}_1q_1^p(1909)$, in notation ${}_1q_1^c(1908) = {}_1q_1^p(1909) = {}_1q_1\ (1909)$. This facilitated the estimation of the missing probabilities as explained below. The mortality data requirements for obtaining the survival component differed from cohort to cohort: the cohort of 1908 demanded the greatest amount of data from age 0 to 25; then the cohort 1909 from age 0 to 24; and so on until the cohort of 1932, only requiring data on infant mortality. To complete the information for the missing years we interpolated single age probabilities between two available probabilities at each age. For example, the single age probabilities from the year 1921 at age 1, ${}_1q_1\ (1921)$, were

known as well as the single age probability in the year 1930 at the same age ${}_1q_1$ (1930), and we assumed a linear trend between the two. The only exception was made for the year of the Spanish flu (1918) which showed greater fluctuations than other years. We estimated all probabilities without the year 1918 and then we compared the estimations for that year with the values available: for the infant mortality; at age 8 from the cohort of 1910; and age 18 for the cohort of 1900. Thus, three ratios were obtained of available overestimated 1918 probabilities, for ages 0, 8, and 18. Those ratios and the mortality age-pattern obtained from our linear estimations for 1918 allowed us to estimate the age-pattern and level of mortality for this year.

The sensitivity analysis conducted shows a minor deviation around 0.03 and lower for $q_x$ between US and Swedish females.

## OSM 3 – Figures for Male Part

*Figure A1. Differences between CAL entropy of individual countries and the selected European average, males 1989−2018*

*Notes: The horizontal dotted dark line represents the selected European populations' average entropy level. The solid, dashed, and dot-dash lines represent the population that crosses the average entropy, and with more and less heterogeneous mortality distribution than the average entropy level, respectively. The standard deviation of the populations selected changed from 0.02 in 1989 to 0.01 in 2018*

*Source: Author's calculation based on HMD data (2023) and Bell and Miller (2005).*

*Figure A2. Decomposition of the male CAL entropy gap between the average and specific populations into longevity and lifespan variation. 1989-2018.*

*Notes: red and blue stand for contributions from changes in lifespan variation and longevity, respectively.*

*Source: Author's calculation based on HMD (2023) and Bell and Miller (2005).*

*Figure A3. Decomposition of the time changes in male CAL entropy gap between the average and specific population across time into average entropy improvements, longevity, and lifespan variation. 1990-2018.*

*Notes: Green, red, and blue stand for contributions from changes in average entropy level, and from lifespan variation and longevity, respectively. Contributions are multiplied by 100.*

*Source: Author's calculation based on HMD (2023) and Bell and Miller (2005).*

**Figure A1. Differences between CAL entropy of individual countries and the average Western European, males 1989–2018**

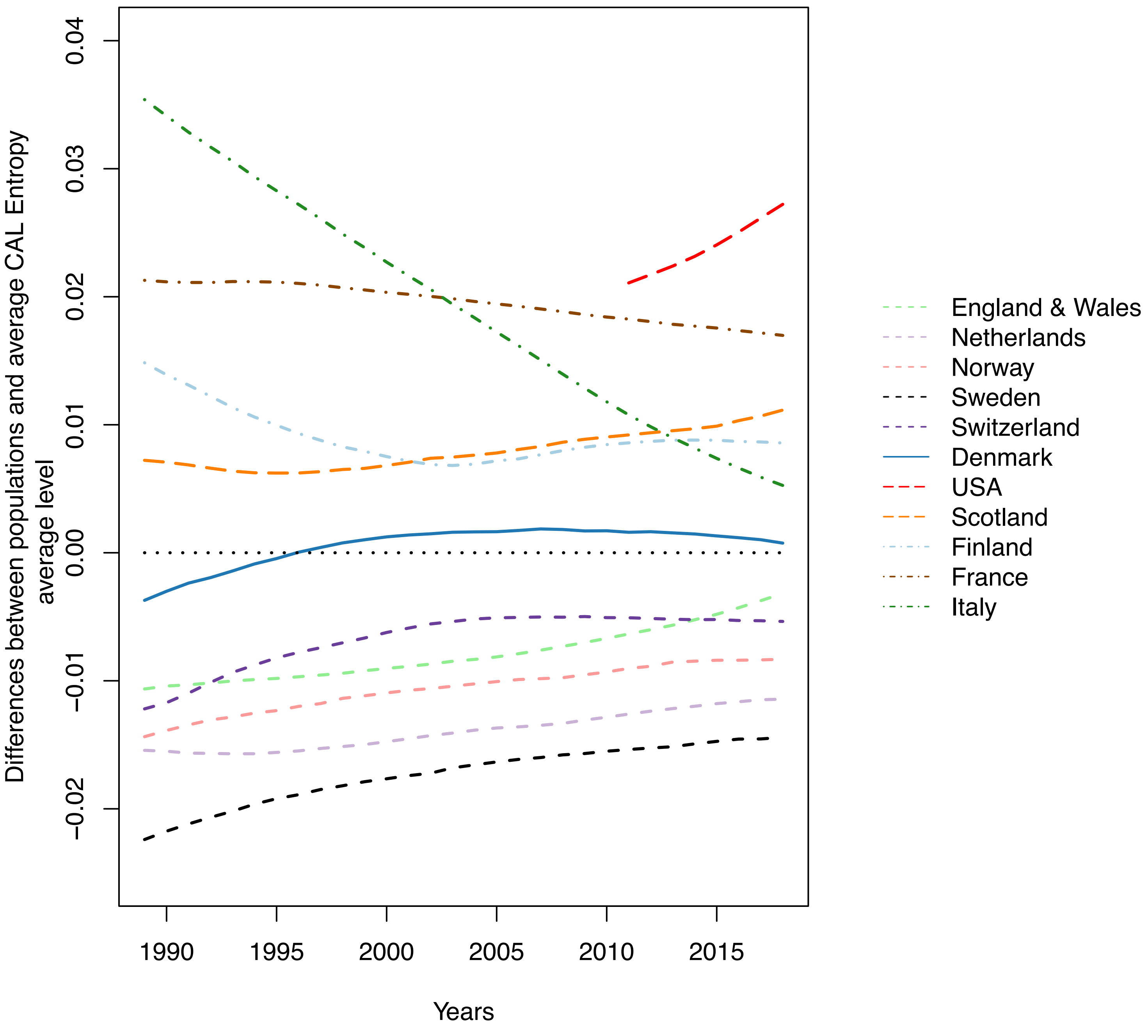

Figure A2. Decomposition of the male CAL entropy gap between the average and specific populations into longevity and lifespan variation. 1989–2018

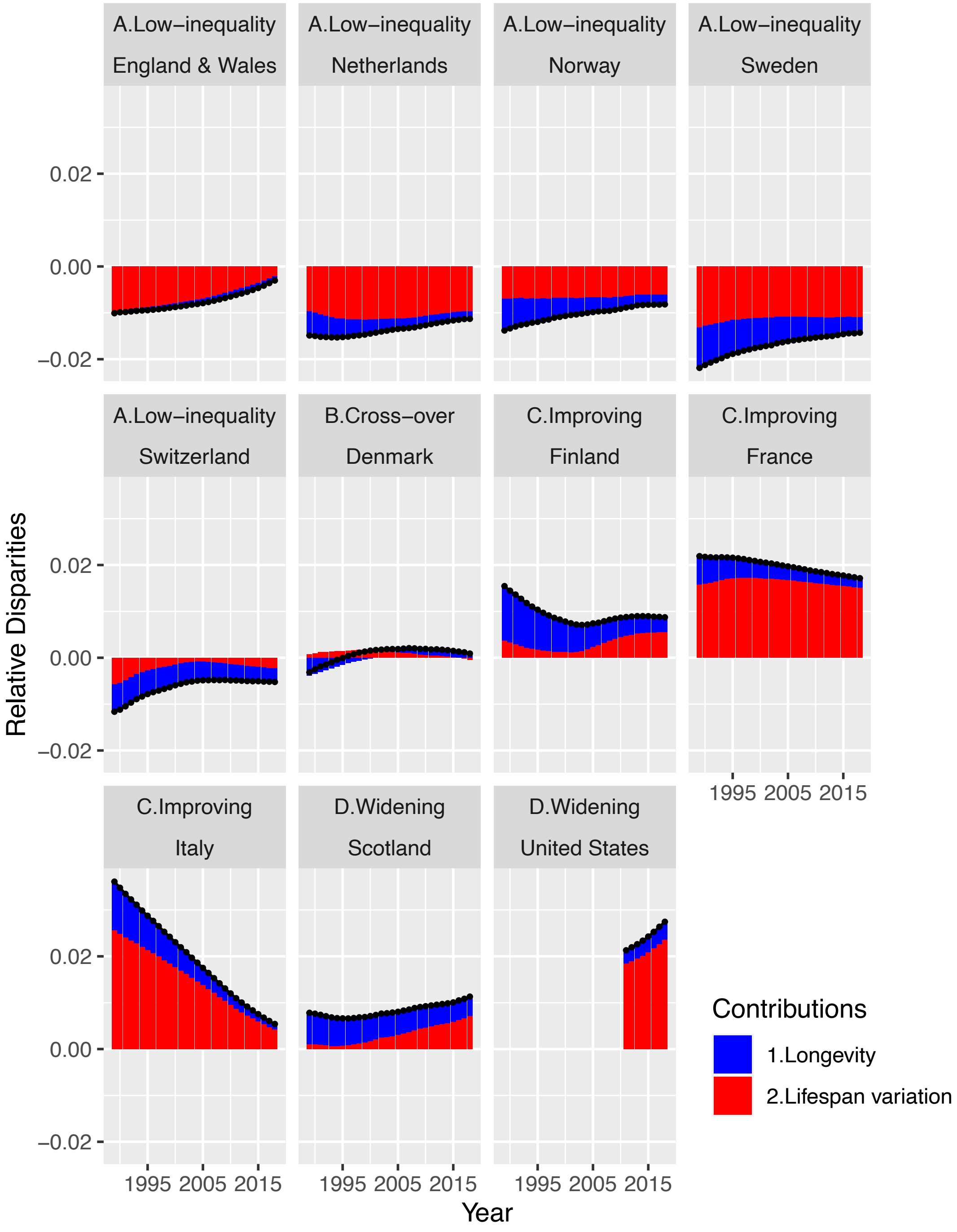

Figure A3. Decomposition of the time changes in male CAL entropy gap between the average and specific population across time into benchmark average entropy, longevity, and lifespan variation. 1990–2018.

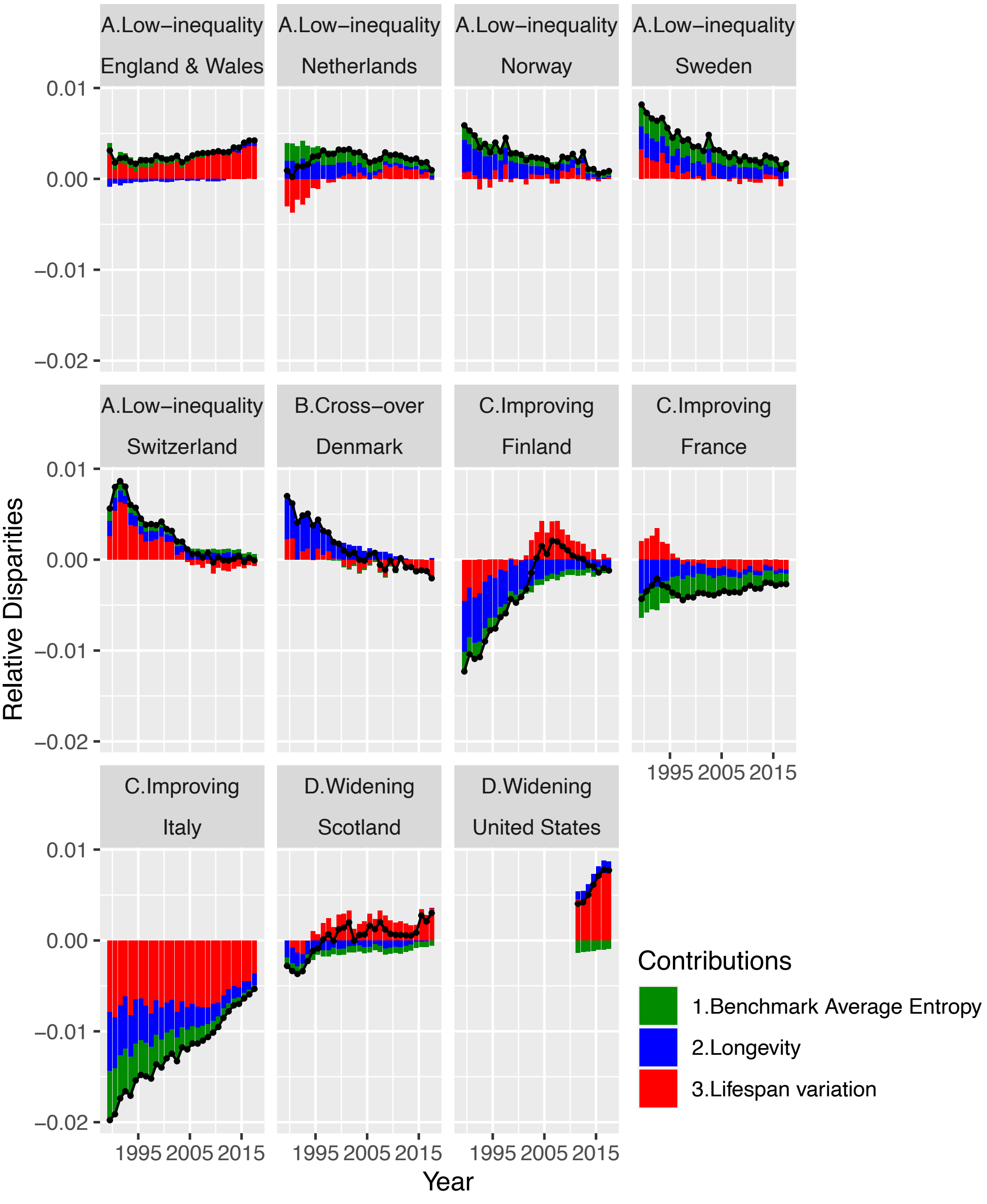

## OSM 4 - $CAL$ Entropy and its sensitivity

Similar to Keyfitz (1977) and Keyfitz and Golini (1975) on the sensitivity of life table entropy to the change in proportional mortality rate across all ages, we illustrate the sensitivity of $CAL$ entropy to the proportional change in age-period-specific mortality rate.

Suppose we have a cohort survival function $\ell_c(x, t-x) = \exp\left(-\int_0^x \mu(x, t-x+a)da\right)$, with $\mu(x, t-x+a)$ being age-cohort-specific mortality rate at time $t$, and we define the proportional change in age-cohort-specific mortality rate across all ages as $\delta$. Therefore, a proportional change in age-cohort-specific mortality will translate into $\mu^*(x, t-x+a) = \mu(x, t-x+a)(1+\delta)$, with * denoting the new age-cohort-specific mortality rate after the proportional change. Based on the exponential property of $\ell_c(x, t-x)$ and $1+\delta$ being a constant, we have:

$$\ell_c^*(x, t-x) = \exp\left(-\int_0^x \mu(x, t-x+a)(1+\delta)da\right) = [\ell_c(x, t-x)]^{1+\delta}. \quad \text{(A9)}$$

We will examine the sensitivity of $CAL$ regarding the proportional change in age-cohort-specific mortality rate by looking at the partial derivative of (A9) respect of proportional change $\delta$. The derivative yields:

$$\frac{d[\ell_c(x,t-x)]^{1+\delta}}{d\delta} = [\ell_c(x, t-x)]^{1+\delta} \ln\,[\ell_c(x, t-x)]. \quad \text{(A10)}$$

Because we want to examine sensitivity concerning the variable $\delta$, we will need to take the Taylor series of $[\ell_c(x, t-x)]^{1+\delta}$ when $\delta$ is in the neighbourhood of zero. This can be justified by $\delta$ usually taking small values and therefore making $[\ell_c(x, t-x)]^{1+\delta}$ a straight line in a dimension regarding $\delta$ (Keyfitz, 1977; Keyfitz & Golini, 1975). By applying Taylor expansion on A10 when $\delta \to 0$, we obtain:

$$[\ell_c(x, t-x)]^{1+\delta} \cong [\ell_c(x, t-x)] + \delta\ell_c(x, t-x)\ln[\ell_c(x, t-x)]. \quad \text{(A11)}$$

Similar to what Keyfitz (1977) has established, and the $\mathcal{H}_{CAL}$ established in the methods section, the equation (A11) can be substituted in the ratio between $CAL^*$ after a proportional change in all age-cohort-specific mortality rate and $CAL$ before such change:

$$\frac{CAL^*}{CAL} = \frac{\int_0^\omega [\ell_c(x,t-x)]^{1+\delta}dx}{\int_0^\omega \ell_c(x,t-x)dx} \cong \frac{\int_0^\omega \ell_c(x,t-x) + \delta\ell_c(x,t-x)\ln[\ell_c(x,t-x)]dx}{\int_0^\omega \ell_c(x,t-x)dx} = 1 - \delta\mathcal{H}_{CAL}. \quad \text{(A12)}$$

## OSM 5 – Age- and cohort-decomposition

*Figure A4. Age− and Cohort−Decomposition of the male CAL-entropy gap between Italian and the Western EU average, males 1903-2013*

*Notes: Each data point in the figure represents the contribution to the differences between males in Italy and Western European average at a given year from a specific cohort up until one specific age. The total value equals the differences in CAL entropy between Italy and Western European average in 2013, as observed in Figure A1. The decomposition was done using the stepwise-replacement algorithm developed by Andreev et al., (2002).*

*Source: Author's calculation based on HMD (2022).*

*Figure A5. Age− and Cohort−Decomposition of changes in the male CAL-entropy gap between Italian and the Western EU average, males 1990-2018*

*Notes: The year on the x-axis represents the mid-point of the changes from the earliest period to the latest period (2004 will be the mid-point between 1990 and 2018). Each data point in the figure represents the contribution to the changes in differences between males in Italy and the Western European average from 1990 to 2018 from a specific cohort up until one specific age. The total value equals the changes in CAL entropy differences between Italy and the Western European average from 1990 to 2018, as observed in Figure A3. The decomposition was done using the stepwise-replacement algorithm developed by Andreev et al., (2002).*

*Source: Author's calculation based on HMD (2022).*

**Figure A4. Age– and Cohort– Decomposition of the male entropy gap between Italian and the Western EU average, males 1903-2013**

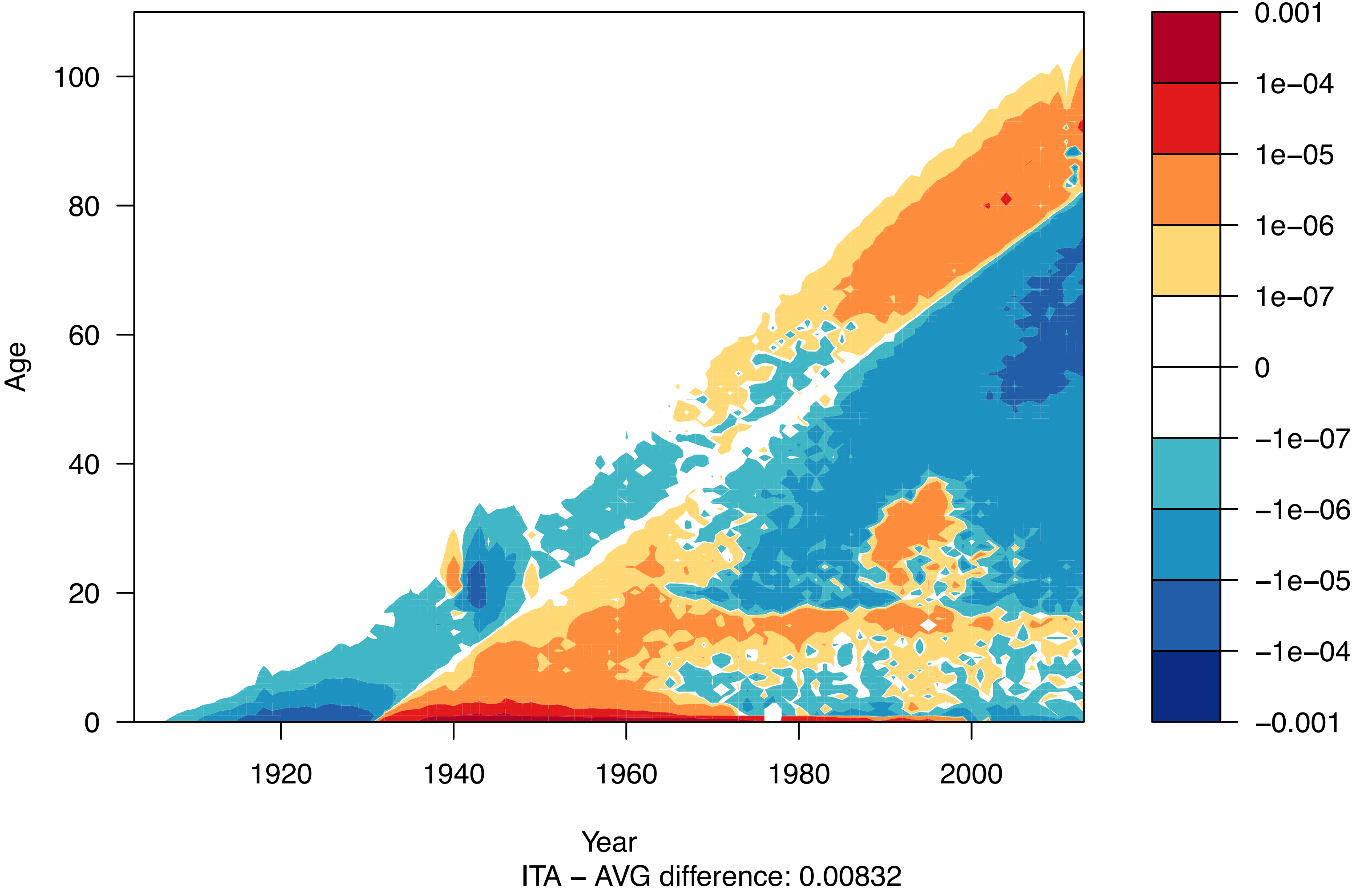

**Figure A5.** ***Age–*** **and** ***Cohort– Decomposition*** **of the male entropy gap** ***across time,*** **between Italian and the Western EU average, males** ***1990–2018***

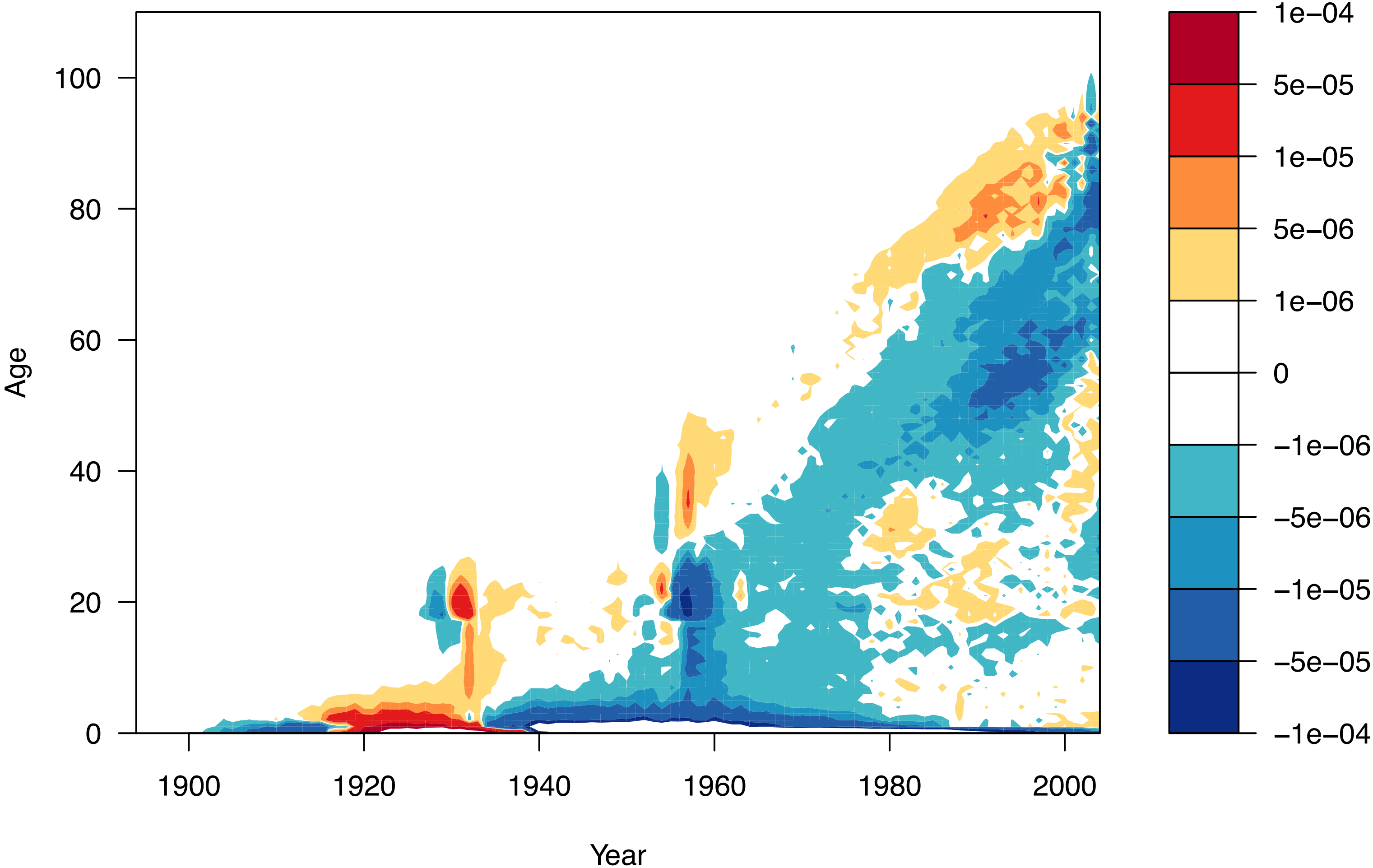


ITA – AVG differences across time: 0.0273

## OSM 6 – Life table entropy surface

The relationship between lifespan variation ($e^{\dagger}$), longevity ($e_0$), and life table entropy ($\mathcal{H}_p$), all from a period perspective, can be seen in the figure below. This figure presents the combinations of lifespan variation and longevity level that return specific entropy values (observed as the lines moving from bottom left to top right with labels starting at 0.1 and reaching 0.34). Also included in this plot are period entropy values calculated from all the male populations in HMD, as of 2018. The pattern for males shows a clear upward trend from higher entropy to lower entropy across time, accompanied by a lower level of lifespan variation and higher longevity through time. However, because life table entropy measures the lifespan variation level with respect to its longevity level, a population with lower levels of longevity and lower levels of lifespan variation could achieve a similar entropy level to a population with higher levels of longevity and lifespan variation. This can also be interpreted as two populations achieving similar concavity in the shape of their respective survival curves. For example, the entropy index for Finnish males in 1985 is 0.17 (with life expectancy of 70.5 and lifespan variation of 11.8), which is approximately the same as the United States male population in 2018 (with life expectancy of 76.5 and lifespan variation of 12.7). This result also relates to Aburto et al., (2020) and Vaupel et al., (2011).

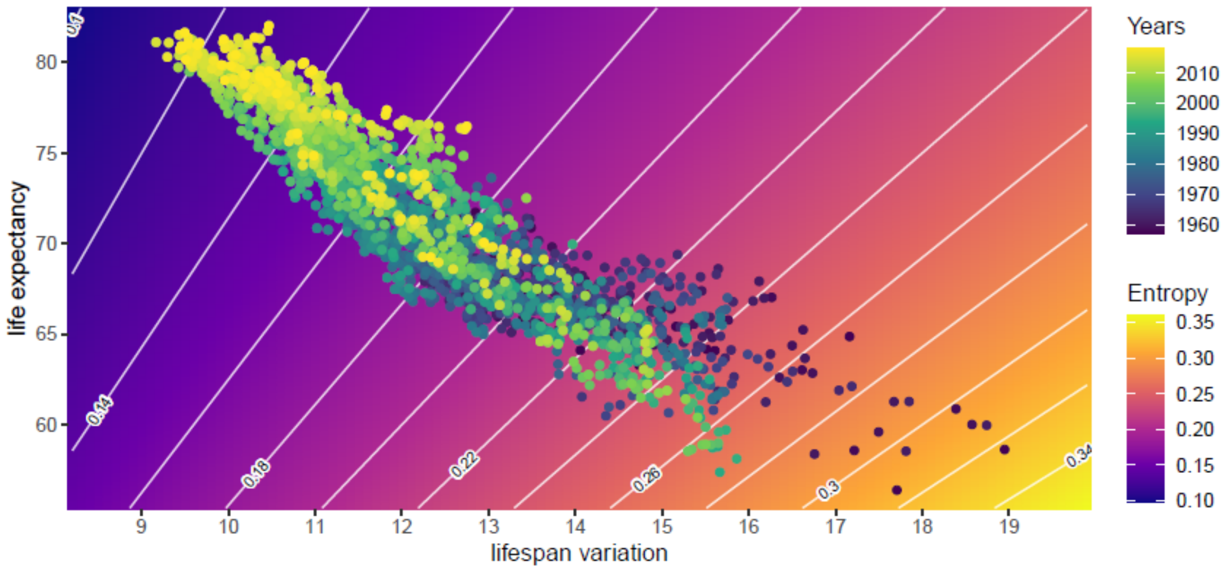


*Life table entropy surface, male, 1957-2018*

*Notes: the x-axis is the level of lifespan variation measure "$e^{\dagger}$", and the y-axis is the level of period life expectancy "$e_0$". The gradient-coloured area and the subscripted isoquant lines represent the degrees of entropy level. The reverse gradient-coloured dots represent the position of all the male populations in HMD at each year from 1957 to 2018 with respect to $e^{\dagger}$ and $e_0$.*

*Source: author's calculation based on HMD (2022).*

**Reference:**

Aburto, J. M., & van Raalte, A. (2018). Lifespan Dispersion in Times of Life Expectancy Fluctuation: The Case of Central and Eastern Europe. Demography, 55(6), 2071-2096. https://doi.org/10.1007/s13524-018-0729-9

Aburto, J. M., Villavicencio, F., Basellini, U., Kjærgaard, S., & Vaupel, J. W. (2020). Dynamics of life expectancy and life span equality. Proceedings of the National Academy of Sciences, 117(10), 5250. https://doi.org/10.1073/pnas.1915884117

Andreev, E. M., Shkolnikov, V. M., & Begun, A. (2002). Algorithm for decomposition of differences between aggregate demographic measures and its application to life expectancies, healthy life expectancies, parity-progression ratios and total fertility rates. Demographic Research, 7(14), 499-522. https://doi.org/10.4054/DemRes.2002.7.14

Bell, F.C. & Miller, M.L. (2005). Social Security Administration. Life Tables for the United States Social Security Area 1900-2100. ACTUARIAL STUDY NO. 120. available at: https://www.ssa.gov/oact/NOTES/as120/TOC.html

Centers for Disease Control and Prevention, CDC (2020). Table 1-1. Live Births, Birth Rates, and Fertility Rates, by Race: United States, 1909-2000. Available at https://www.cdc.gov/nchs/data/statab/t001x01.pdf

Guillot, M. (2003). The cross-sectional average length of life (CAL): A cross-sectional mortality measure that reflects the experience of cohorts. Population Studies, 57(1), 41-54. https://doi.org/10.1080/0032472032000061712

HMD. (2022). HMD (Human Mortality Database). University of California, Berkeley (USA), and Max Planck Institute for Demographic Research (Germany). Available: http://www.mortality.org

Keyfitz, N. (1977). What difference would it make if cancer were eradicated? An examination of the taeuber paradox. Demography, 14(4), 411-418. https://doi.org/10.2307/2060587

Keyfitz, N., & Golini, A. (1975). Mortality Comparisons: The Male—Female Ratio. Genus, 31(1/4), 1-34. http://www.jstor.org/stable/29788095

Nepomuceno, M. R., Cui, Q., van Raalte, A., Aburto, J. M., & Canudas-Romo, V. (2021). The Cross-sectional Average Inequality in Lifespan (CAL†): A Lifespan Variation Measure That Reflects the Mortality Histories of Cohorts. Demography. https://doi.org/10.1215/00703370-9637380

Preston, S., Heuveline, P., & Guillot, M. (2001). Demography: measuring and modeling population processes. Malden, MA: Blackwell Publishers.

Vaupel, J. W., & Canudas-Romo, V. (2003). Decomposing change in life expectancy: A bouquet of formulas in honor of Nathan Keyfitz's 90th birthday. Demography, 40(2), 201-216. https://doi.org/10.1353/dem.2003.0018

Vaupel, J. W., Zhang, Z., & van Raalte, A. A. (2011). Life expectancy and disparity: an international comparison of life table data. BMJ Open, 1(1), e000128. https://doi.org/10.1136/bmjopen-2011-000128
